# Atorvastatin-associated liver injury: a structured narrative review of reporting signals, clinical phenotypes, and hepatic safety, 2023–2026

**DOI:** 10.3389/fphar.2026.2003438

**Published:** 2026-10-30

**Authors:** Hao Zhang, PanLi Zhao, Hua Huang, YuQi Zeng

**Affiliations:** Department of Pharmacy, Chengdu Seventh People’s Hospital (Affiliated Cancer Hospital of Chengdu Medical College), Chengdu, China

**Keywords:** atorvastatin, autoimmune hepatitis, drug-induced liver injury, hepatotoxicity, pharmacovigilance

## Abstract

**Background:**

Atorvastatin is widely prescribed, yet its hepatic safety remains debated: randomized trials and biopsy-proven cohorts indicate acceptable safety, whereas pharmacovigilance data and case reports describe injury ranging from asymptomatic transaminase elevation to acute liver failure.

**Methods:**

PubMed was searched for records combining atorvastatin terms with liver-injury concepts, published 1 July 2023 to 31 July 2026; records were screened in duplicate and assigned to one of four outcome domains—biochemical liver test abnormality, suspected drug-induced liver injury (DILI), clinically significant or severe DILI, and acute liver failure—and were not combined across domains. This is a structured narrative review, not a systematic review: only PubMed was searched and comprehensive retrieval is not claimed.

**Results:**

Of 119 records, 57 were included in the synthesis: 31 directly addressed atorvastatin and liver injury and 26 addressed statin hepatic safety or statin use in chronic liver disease. Among 7,779 statin-associated drug-induced liver injury (DILI) cases in FAERS (2004–2023), atorvastatin accounted for 53.48%, with a reporting odds ratio of 3.09. In 1 211 reported Chinese cases of statin-associated liver injury, the odds of severe injury were 1.77-fold higher with atorvastatin than with rosuvastatin, and 2.08-fold higher with combination therapy than with statin monotherapy. Phenotypes included hepatocellular, cholestatic and mixed injury, drug-induced autoimmune hepatitis (DIAIH) and hyperacute liver failure, usually within 2–3 months. Rechallenge reproduced injury in 12 of 16 cases, with median latency shortened from 27 to 11 days, whereas switching to a hydrophilic statin was usually tolerated. Mechanisms involve mitochondrial dysfunction, oxidative stress, CYP3A-mediated metabolic activation, bile-acid transporter inhibition and pharmacogenomic variation. Conversely, in MASLD and cirrhosis, atorvastatin did not worsen liver injury and reduced liver-related events.

**Conclusion:**

Severe atorvastatin-associated liver injury is rare but recognizable. Structured monitoring, causality assessment and injury phenotyping are essential, same-drug rechallenge should be avoided, and hydrophilic statins preferred. Statin therapy should not be withheld in chronic liver disease.

## 1 Introduction

Statins are the cornerstone of primary and secondary prevention of atherosclerotic cardiovascular disease. Atorvastatin, with its potent low-density lipoprotein cholesterol (LDL-C)-lowering effect, long half-life, and extensive evidence base, has long ranked among the most prescribed lipid-lowering agents worldwide. As the treated population has expanded and treatment intensity has increased, its hepatic safety has remained incompletely resolved (Penedones et al., 2026; Li et al., 2025).

This uncertainty has historical roots. Early trials and post-marketing surveillance treated transaminase elevation as the principal signal of statin hepatotoxicity, prompting several guideline generations to recommend frequent routine liver monitoring and to list chronic liver disease as a relative contraindication. Subsequent work showed that statin-associated transaminase elevation is usually asymptomatic, dose-dependent and reversible, and is mechanistically and prognostically distinct from true drug-induced liver injury (DILI) (Abdel-Rasol and El-Sayed, 2026). From the 2010s onwards, studies further suggested that statins are not only safe in MASLD, chronic viral hepatitis and cirrhosis, but may confer liver-related benefit (Liou et al., 2026; Glal et al., 2026; Wu et al., 2023).

At the same time, pharmacovigilance data and case reports have continued to generate a consistent signal of hepatic injury for atorvastatin, with a phenotypic spectrum broader than originally appreciated. Beyond hepatocellular and cholestatic injury, drug-induced autoimmune hepatitis (DIAIH) has become a focus of reporting over the past 3 years (Serrano-Pérez et al., 2026; Loeza-Suárez et al., 2025; Patel et al., 2024; Tse et al., 2023); single cases have developed hyperacute liver failure within 24 h of starting 80 mg (AlTurki et al., 2026); and the proportion of patients experiencing recurrence after rechallenge appears higher than previously estimated (Bertin et al., 2026; Singh et al., 2025).

The period 2023–2026 has been one of rapid evidence accumulation. Large real-world studies based on FAERS, EudraVigilance, MID-NET and TriNetX have provided the largest risk-quantification datasets to date; randomized trials and biopsy-proven cohorts have supplied high-grade safety evidence in chronic liver disease; and mechanistic work on metabolic activation, drug transporters and pharmacogenomics has extended our understanding to the molecular level (Abdel-Rasol and El-Sayed, 2026; Yuan et al., 2026; Guan et al., 2026; Nabil et al., 2026).

We therefore systematically reviewed the PubMed literature of the past 3 years to characterize the epidemiology, clinical phenotypes, risk factors, mechanisms, and clinical management of atorvastatin-associated liver injury, and to identify points of disagreement and research gaps that should inform both clinical decisions and future studies.

## 2 Methods

### 2.1 Search strategy

This article is a structured narrative review. It is not a systematic review and does not claim comprehensive retrieval: only PubMed was searched and no protocol was registered. We searched PubMed using a combination of Medical Subject Headings (MeSH) and free-text terms. The drug component used ‘atorvastatin’. The outcome component used the free-text terms ‘liver injury’, ‘liver injuries’, ‘hepatotoxicity’, ‘hepatotoxic’, ‘drug-induced liver injury’, ‘liver damage’, ‘hepatic injury’, ‘liver toxicity’, ‘elevated transaminases’, ‘transaminase elevation’, ‘liver enzyme’ and ‘liver enzymes’ in Title/Abstract, together with the MeSH terms “Chemical and Drug Induced Liver Injury”, “Hepatotoxicity”, “Liver Failure, Acute” and “Liver Diseases”. The search was restricted to publication dates from 1 July 2023 to 31 July 2026 and sorted by publication date in descending order. Only PubMed was searched; Embase, Web of Science, Scopus and the Cochrane Database of Systematic Reviews were not searched, and the consequences of this restriction are set out in the Limitations.

### 2.2 Eligibility criteria

Records were eligible if they (1) concerned atorvastatin or reported data on atorvastatin within a multi-statin analysis; (2) addressed hepatic safety, mechanisms of liver injury, liver enzyme changes, or statin use in chronic liver disease; (3) were published within the defined window; and (4) had retrievable bibliographic and abstract information. Records were excluded if atorvastatin served only as background therapy or a comparator without any liver-related outcome, if the topic was clearly unrelated to drug-induced liver injury (for example, oncology, infectious disease, or aquatic toxicology), or if they were duplicate or incomplete.

### 2.3 Data extraction and synthesis

Two authors independently screened titles and abstracts against the pre-specified criteria; agreement was quantified using Cohen’s kappa (kappa = 0.73, 95% CI 0.61–0.85; observed agreement 86.6%). The 16 records on which the two screeners disagreed were resolved by a third author, giving 58 records for synthesis; one of these was excluded on author review, leaving 57 records for synthesis. The same two authors extracted data independently onto a standardised form. We extracted bibliographic data (PMID, DOI, journal, year, authors), study design and sample size, exposure and outcome definitions, and principal results with effect estimates. Records were classified into three tiers: those directly investigating atorvastatin and liver injury; those addressing statin hepatic safety or statin use in chronic liver disease; and those merely mentioning atorvastatin. The first two tiers were synthesized. Within the synthesized records, findings were then assigned to one of four outcome domains—biochemical liver test abnormality; suspected DILI; clinically significant or severe DILI; and acute liver failure—and were not combined across domains, because these outcomes differ fundamentally in clinical meaning. Because of substantial heterogeneity in design, populations and outcome definitions, we used narrative synthesis rather than quantitative pooling.

## 3 Results

### 3.1 Characteristics of the retrieved literature

The search identified 120 records; one did not retrieve as an eligible indexed article record, leaving 119 records for screening, all with abstracts (Tables 1, 2). By publication year, 14 (11.8%) appeared in 2023, 25 (21.0%) in 2024, 39 (32.8%) in 2025 and 41 (34.5%) in 2026, indicating a steadily increasing research interest.

**TABLE 1:** Characteristics of the retrieved literature.

Item	Category	Records	Proportion
Publication year	2023	14	11.8%
	2024	25	21.0%
​	2025	39	32.8%
​	2026	41	34.5%
Study type	Original research article	44	37.0%
	Basic or experimental study	39	32.8%
​	Case report	14	11.8%
​	Observational or comparative study	7	5.9%
​	Randomized controlled trial	6	5.0%
​	Review	5	4.2%
​	Meta-analysis	3	2.5%
​	Clinical trial	1	0.8%
Relevance	Included in the evidence synthesis: directly addresses atorvastatin and liver injury	31	26.1%
	Included in the evidence synthesis: statin hepatic safety or statin use in chronic liver disease	26	21.8%
​	Not included: mentions atorvastatin only	62	52.1%
Total	—	119	100%

**TABLE 2:** Summary of key studies.

Study (year)	Design/data source	Sample size	Principal findings
Zhou et al. (2025)	FAERS pharmacovigilance, 2004–2023	7,779 statin-associated DILI cases	Atorvastatin accounted for 53.48%; ROR 3.09, second only to fluvastatin (6.90)
Wang et al. (2024); Ogura and Shiraishi (2025)	FAERS pharmacovigilance, 2004–2024 Q1	14,511 hepatic disorder events	Positive signals for all seven statins; atorvastatin showed the strongest signal for cholestatic pruritus and abnormal bilirubin conjugation
Yuan et al. (2026)	FAERS + EudraVigilance	104,798 reports	597 significant signals; rhabdomyolysis and DILI were major designated medical events; liver failure, hepatocellular injury or death in 1,833 cases (4.91%)
Su et al. (2024); Wang et al. (2024)	Shandong ADR monitoring database, China, 2013–2021	1,211 cases of statin-induced liver injury (157 severe)	Atorvastatin conferred a 1.77-fold higher risk of severe injury than rosuvastatin; combination therapy 2.08-fold higher than monotherapy
Bertin et al. (2026)	French pharmacovigilance database, rechallenge study	26 cases	Positive rechallenge in 12 of 16 same-drug cases; median latency shortened from 27 to 11 days; hydrophilic statins usually tolerated
Chen et al. (2025); Abdel-Rasol et al. (2026)	Target trial emulation, Tianjin, China, 2013–2022	25,728 with elevated liver enzymes; 18,270 with normal liver enzymes	In the elevated-liver-enzyme group, atorvastatin vs. rosuvastatin: 1-year all-cause mortality HR 1.29; no difference in the normal-liver-enzyme group
Chun et al. (2025); Li et al. (2024)	Common data model, 14 hospitals, South Korea, 19 million patients	3,655–11 799 per cohort	Pravastatin 40 mg and atorvastatin 10 mg were comparably effective; abnormal AST 2.35% vs. 3.37%
Glal et al. (2026)	Randomized, double-blind, placebo-controlled trial	100 patients with decompensated cirrhosis	Recurrent decompensation 36% vs. 72% (HR 0.50); hepatorenal syndrome 0% vs. 20%; transaminase elevations transient
Kronborg et al. (2023); Kawas et al. (2026)	Randomized, double-blind, placebo-controlled trial	78 patients with cirrhosis and portal hypertension	Safe, but no reduction in mortality, liver-related complications or hepatic venous pressure gradient
Liou et al. (2026)	Prospective biopsy-proven MASLD cohort	215 patients (median treatment duration 63 months)	Statin use did not worsen liver enzymes or fibrosis, including in advanced fibrosis
Ma et al. (2026); Tsui et al. (2026)	NLP-based pharmacovigilance in hospitalized older adults	109,263 patients; 3,227 drug combinations	Atorvastatin plus piperacillin/tazobactam produced a significant hepatotoxic interaction signal
Eslami et al. (2025); Karachaliou et al. (2026)	Rat model of NAFLD	56 animals	Atorvastatin mainly improved hepatocyte ballooning and lobular inflammation; limited effect on liver enzymes
Guan et al. (2026)	*In vitro* metabolic activation plus *in vivo* mouse study	—	First demonstration that CYP3A-mediated quinone-imine intermediates form covalent protein adducts
Nabil et al. (2026)	Mouse hepatic transcriptomics and ATAC-seq	—	Atorvastatin acts as a selective PXR activator and does not induce hepatic steatosis
Abdel-Rasol et al. (2026); Al-Shalchi and Mohammad (2024)	Rat study	40 animals	Coenzyme Q10 restored CYP3A1 activity from ∼29% to ∼90% of control and attenuated liver injury
Elsiad et al. (2025); Marchianò et al. (2023)	Rat study	—	Liraglutide attenuated atorvastatin-induced hepatotoxicity *via* Nrf2/SOD and autophagy pathways
Ramandi et al. (2023); Zhang et al. (2024)	Randomized controlled trial, 5-year follow-up	1,596 participants stratified by MAFLD	Atorvastatin-containing polypill reduced MACE (HR 0.56) and cardiovascular mortality (HR 0.41) in MAFLD, with greater benefit than in the general population
Dautović et al. (2023); Chun et al. (2025)	Comparative HepG2 cell study	—	Cytotoxicity and reactive oxygen species production were greater with atorvastatin and simvastatin than with rosuvastatin, reflecting differences in lipophilicity and cellular accumulation

By design, there were 44 original research articles, 39 basic or experimental studies, 14 case reports, 7 observational or comparative studies, 6 randomized controlled trials, 5 reviews, 3 meta-analyses and 1 clinical trial. Basic studies and case reports together accounted for nearly half of the corpus, indicating that the field remains dominated by mechanistic exploration and descriptions of rare events, with relatively few large prospective studies.

By relevance, 31 records (26.1%) directly investigated atorvastatin and liver injury, 26 (21.8%) addressed statin hepatic safety or statin use in chronic liver disease, and the remaining 62 mentioned atorvastatin only in passing. The first two groups were included in the evidence synthesis (n = 57); the third group was retrieved by the search but was not synthesised.

### 3.2 Epidemiological landscape and pharmacovigilance signals

Overall incidence: Pooled analyses place the incidence of statin-associated liver enzyme abnormalities at approximately 0.2%–0.6%. A pooled analysis of Chinese clinical trials conducted between 1992 and 2023 reported an incidence of liver-related adverse drug reactions of 0.4%–0.6% (Tsui et al., 2025), whereas a meta-analysis of 41 randomized controlled trials including 64,728 participants reported a pooled incidence of transaminase elevation of 0.201% (95% CI 0.004%–0.398%) among users of high-dose statins (Li et al., 2025). Injury reaching clinical significance is rarer still and is generally regarded as an idiosyncratic reaction (Abdel-Rasol and El-Sayed, 2026).

Reporting share and signal strength for atorvastatin: FAERS identified 7,779 cases of DILI reported between 2004 and 2023 in which a statin was the primary suspected drug. Atorvastatin accounted for 53.48% of these, far exceeding rosuvastatin (20.44%) and simvastatin (19.46%). Signal strength ranked as fluvastatin (ROR 6.90, 95% CI 5.89–8.10) > atorvastatin (ROR 3.09, 95% CI 2.99–3.19) > simvastatin (2.96) > lovastatin (2.77) > rosuvastatin (2.27) > pravastatin (2.07). Among DILI cases, 45.43% were older than 65 years and 39.95% required hospitalization; mortality was highest for simvastatin-associated cases (12.15%) (Zhou et al., 2025). A head-to-head FAERS analysis restricted to patients with a documented indication of hypercholesterolaemia (2004–2024), using 21 pairwise comparisons with Bonferroni correction, found a heterogeneous rather than a consistent ordering: relative to atorvastatin, five statins showed significantly higher adjusted reporting odds ratios for gastrointestinal disorders, and five showed significantly lower ratios for metabolic disorders (Ogura and Shiraishi, 2025).

Phenotype-level signals: A separate analysis covering FAERS from the first quarter of 2004 to the first quarter of 2024 identified 14,511 hepatic disorder events and 148 positive signals; all seven statins produced positive signals. Atorvastatin showed the strongest signal strength for cholestatic pruritus and abnormal bilirubin conjugation, while autoimmune hepatitis and DILI produced positive signals across multiple statins (Wang et al., 2024).

Cross-database corroboration: An analysis combining FAERS (2004–2024) and EudraVigilance (2002–2024) included 104,798 reports related to atorvastatin calcium (67,498 and 37,300, respectively) and identified 597 significant signals using four algorithms. Rhabdomyolysis and drug-induced liver injury were major designated medical events in both databases. In EudraVigilance, liver failure, hepatocellular injury and death were additionally identified as key serious outcomes in 1,833 cases (4.91%). Older patients (34.12% aged ≥ 65 years) and women (49.83%) warrant specific attention (Yuan et al., 2026).

Chinese population data: A study based on the Shandong Provincial Adverse Drug Reaction Monitoring Center and hospital records screened 1,391 reports, of which 1,211 met the criteria for statin-induced liver injury, including 157 severe cases. Multivariable analysis identified concomitant drug use and the specific statin as independent determinants of severe injury: atorvastatin conferred a 1.77-fold higher risk of severe liver injury than rosuvastatin, and combination therapy a 2.08-fold higher risk than monotherapy (Su et al., 2024). Pooled Chinese trial data likewise ranked pitavastatin (5.36%) and atorvastatin (1.819%) highest for liver-related adverse drug reactions (Tsui et al., 2025; Tsui et al., 2026).

Drug–drug interaction signals: A natural language processing pharmacovigilance study of 109,263 hospitalized older adults evaluating 3,227 drug combinations identified 111 hepatotoxicity-associated interaction signals, among which atorvastatin combined with piperacillin/tazobactam showed a marked risk increase (additive interaction 0.52, multiplicative interaction 1.88), corroborated by animal experiments and sensitivity analyses (Ma et al., 2026).

Generic versus brand statins: Using the Japanese MID-NET database, new users of six statins between 2014 and 2022 were identified. The adjusted hazard ratio (aHR) for liver dysfunction with generic versus brand atorvastatin was 2.08 (95% CI 1.20–3.63). However, with progressively shorter follow-up the aHR approached unity (1.74 at 360 days, 1.65 at 180 days, 1.49 at 90 days and 1.33 at 30 days), suggesting that the apparent difference is influenced by unmeasured confounding and does not establish a genuine safety difference between generic and brand products (Maruyama et al., 2026).

### 3.3 Clinical phenotypes, latency, and prognosis

Biochemical liver test abnormality is the most common presentation. Aminotransferase elevation of this kind is usually dose-dependent and resolves with continued use or dose reduction. Suspected DILI. In a French pharmacovigilance series of 26 cases of atorvastatin-associated liver injury, the cholestatic pattern was most frequent (42.3%), followed by hepatocellular (34.6%) and mixed (23.1%) (Bertin et al., 2026). Clinically significant or severe DILI. At least six case reports or series published in the last 3 years describe atorvastatin-associated DIAIH or autoimmune hepatitis, with lymphocyte and plasma cell infiltration on histology. These cases must be distinguished from idiopathic autoimmune hepatitis and generally respond well to corticosteroids (Serrano-Pérez et al., 2026; Loeza-Suárez et al., 2025; Patel et al., 2024; Tse et al., 2023; Khadijat-Ul-Kurba et al., 2025). Acute liver failure. A man in his seventies developed hyperacute hepatic failure within 24 h of starting atorvastatin 80 mg for secondary stroke prevention; after exclusion of alternative causes, DILI was diagnosed and the patient improved rapidly on withdrawal (AlTurki et al., 2026). Several reports describe liver injury occurring together with myopathy and rhabdomyolysis, including one case that worsened on rechallenge, with both the Roussel Uclaf Causality Assessment Method (RUCAM) and the Revised Electronic Causality Assessment Method (RECAM) indicating a highly probable causal role for atorvastatin (Singh et al., 2025; Kuraning et al., 2025; Kashyap et al., 2023). In an analysis of 60 published DILI case reports from the past decade, most reactions occurred within 2.3 months of treatment initiation (Misra et al., 2026); in the French pharmacovigilance series the median time to onset of the first episode was 27 days (IQR 4–43 days) (Bertin et al., 2026). Notably, cases of cholestatic hepatitis with unusually prolonged latency have also been described, indicating that strict reliance on classical temporal criteria may lead to under-recognition or underestimation of causality (Ruiz et al., 2026). The overall prognosis is favorable. In the 60-case analysis, drug withdrawal was the principal intervention (93%), 87% of patients recovered fully, 3% died, and 78% required hospitalization (Misra et al., 2026). In a retrospective study of 1,167 DILI cases in China, 23.99% were cured, 59.21% improved, 16.20% did not improve, and 0.60% died (Ma et al., 2024). Autoimmune-like presentations usually require corticosteroids and may require prolonged treatment (Serrano-Pérez et al., 2026; Patel et al., 2024). Biochemical liver test abnormality. Isolated elevation of gamma-glutamyl transferase (GGT) with normal transaminases is not sufficient to diagnose statin hepatotoxicity. In one case, persistent GGT elevation above 100 U/L developed during atorvastatin therapy and persisted after withdrawal; evaluation ultimately attributed it to uncontrolled cardiometabolic risk factors. The authors proposed a stepwise assessment framework to avoid unnecessary discontinuation of a necessary statin (Yang et al., 2026).

### 3.4 Factors associated with reported liver injury

In FAERS, 45.43% of DILI cases occurred in patients older than 65 years, and for atorvastatin, simvastatin, pravastatin and fluvastatin the signal was stronger in patients aged ≥65 years than in younger patients (Zhou et al., 2025). In the Chinese DILI study, female sex was independently associated with DILI (odds ratio [OR] 1.897, P < 0.001) (Ma et al., 2024), and women also predominated in the Eudra Vigilance dataset (Yuan et al., 2026). Dose is related to hepatic findings in three ways that should not be conflated: elevation of aminotransferases is dose-dependent, predictable and usually reversible; idiosyncratic DILI is rare and largely dose-independent within the therapeutic range; and severe liver injury is a rare consequence of the latter rather than a direct extension of the former. Meta-analytic data show higher rates of transaminase elevation with simvastatin 40 mg and atorvastatin 80 mg than with other regimens in patients with acute coronary syndrome or acute stroke (Li et al., 2025); the case of hyperacute liver failure also occurred at 80 mg (AlTurki et al., 2026). The odds of severe liver injury were approximately 2.08-fold higher with combination therapy than with statin monotherapy (Su et al., 2024), and 75% of cases in a DILI case series involved multiple medications (Misra et al., 2026). Because atorvastatin is metabolized by CYP3A4, co-administration with CYP3A4 inhibitors or with drugs sharing the same transporters increases exposure and may increase the likelihood of injury (Abdel-Rasol and El-Sayed, 2026). MASLD and MASH, together with obesity, substantially alter atorvastatin pharmacokinetics. Simulations using a permeability-limited multicompartment liver model predicted more than a 250% increase in peak plasma concentration and area under the curve in MASH, with unbound hepatocellular exposure increasing by up to 127% in the periportal region (Murphy et al., 2026). In cirrhosis, model-based simulations predict substantially higher exposure with increasing disease severity, corresponding to hypothetical dose reductions of approximately 70%, 84% and 92% for Child–Pugh classes A, B and C. These are model-derived estimates that have not been validated against clinical outcomes; they are not dosing recommendations (Galal et al., 2026). In a prospective multi-ethnic cohort of 675 patients in the United Arab Emirates, carriers of the ABCG2 rs2231142 variant receiving rosuvastatin had an approximately threefold higher risk of liver enzyme elevation, while carriers of SLCO1B1 rs4149056 receiving atorvastatin had an approximately twofold higher risk of statin-associated muscle symptoms (Alqasrawi et al., 2025). CYP3A4/5 and UGT1A1 polymorphisms likewise modulate atorvastatin disposition and susceptibility (Abdel-Rasol and El-Sayed, 2026), and CYP3A4\*1B and CYP3A5\*3 single-nucleotide polymorphisms influence the response to high-intensity statin therapy (Maslub et al., 2024). In a prospective cohort of 145 postmenopausal women treated with atorvastatin or rosuvastatin for 16 weeks, the SLCO1B1 c.521T>C variant was not associated with the lipid-lowering response but was strongly associated with adverse effects (odds ratio 7.4, 95% CI 2.1–26.7 for the T/C genotype), whereas vitamin D status was associated with neither; the outcome was tolerability rather than clinically significant liver injury (Marušić et al., 2026).

### 3.5 Mechanistic pathways

Recent work supports a multi-component, interacting mechanistic model (Figure 1; Table 3).

**FIGURE 1:**
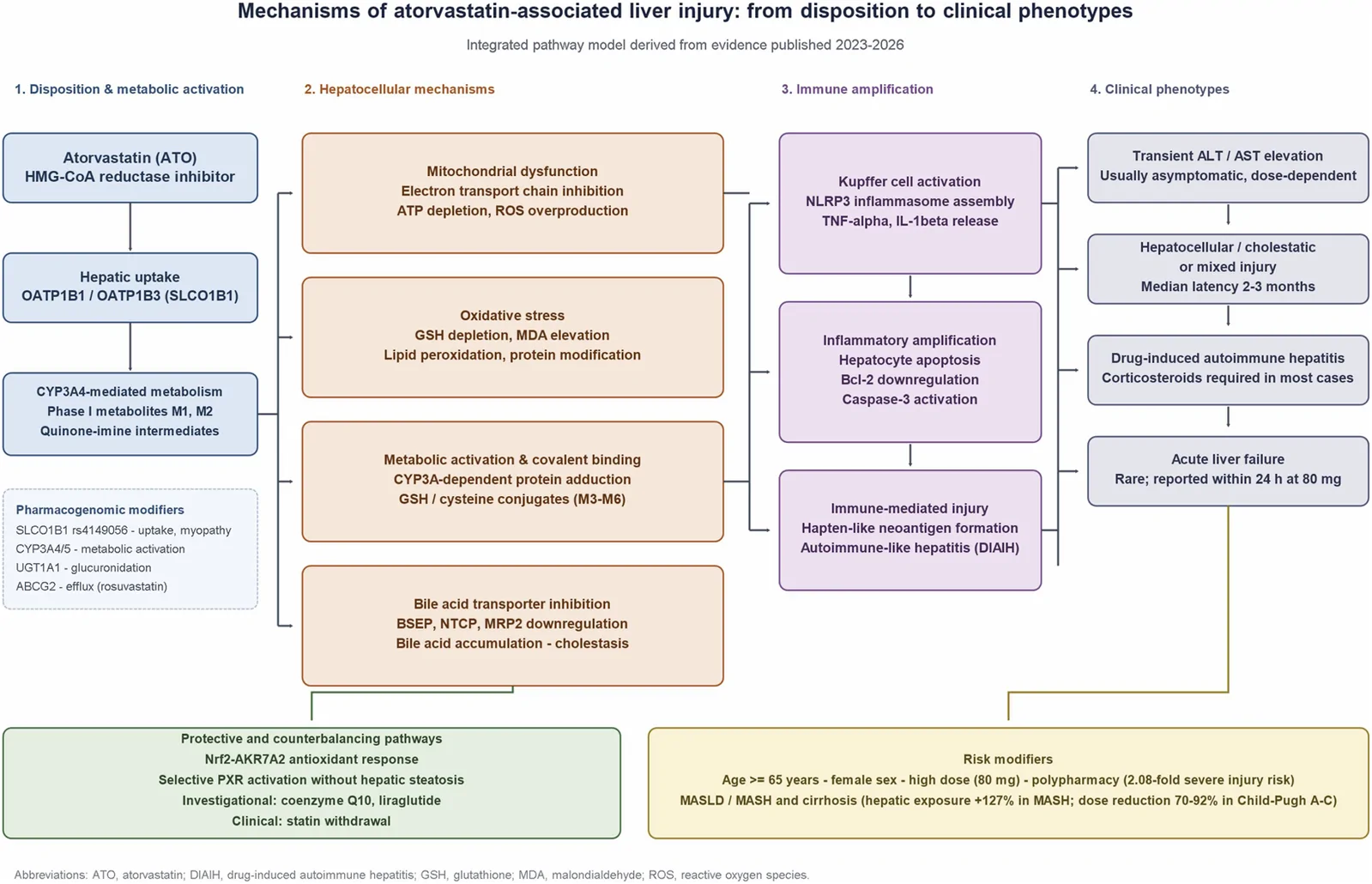
Mechanisms proposed for atorvastatin-associated liver injury.

**TABLE 3:** Mechanistic pathways and supporting evidence.

Mechanistic component	Key molecules/pathways	Corresponding clinical phenotype	Evidence
Mitochondrial dysfunction	Electron transport chain inhibition, ATP depletion, ROS accumulation	Hepatocellular injury, transaminase elevation	Review; animal study (Abdel-Rasol and El-Sayed, 2026; Maslub et al., 2024)
Oxidative stress	Glutathione depletion, malondialdehyde elevation, lipid peroxidation	Dose-dependent liver enzyme elevation	Mouse study (Maslub et al., 2024); cell study (Marušić et al., 2026)
Metabolic activation	CYP3A-mediated quinone-imine formation, covalent protein adduction	Idiosyncratic liver injury	*In vitro* and *in vivo* study (Guan et al., 2026)
Bile-acid transporter inhibition	BSEP, NTCP and MRP2 inhibition	Cholestatic hepatitis	Review (Abdel-Rasol and El-Sayed, 2026); case reports[ (Bertin et al., 2026; Misra et al., 2026)]
Inflammatory and immune activation	Kupffer cells, NLRP3 inflammasome, TNF-α, IL-1β	Drug-induced autoimmune hepatitis	Review (Abdel-Rasol and El-Sayed, 2026); case reports (Serrano-Pérez et al., 2026; Loeza-Suárez et al., 2025; Tse et al., 2023)
Pharmacogenomics	SLCO1B1, CYP3A4/5, UGT1A1, ABCG2	Inter-individual susceptibility	Prospective cohort (Galal et al., 2026); review (Abdel-Rasol and El-Sayed, 2026)
Altered pharmacokinetics	Increased exposure in MASH and cirrhosis	Higher risk in metabolic liver disease	PBPK models (Yang et al., 2026; Murphy et al., 2026)
Protective pathways	Nrf2–AKR7A2, ligand-specific PXR activation	Limitation of injury; hepatic safety	Cell study (Marušić et al., 2026); mouse study (Nabil et al., 2026)

The mechanisms described in this section are derived from experimental systems unless otherwise stated, and do not by themselves establish that these pathways operate in patients. Atorvastatin inhibits mitochondrial electron transport chain complexes, causing ATP depletion and excessive reactive oxygen species (ROS) generation, which in turn drives lipid peroxidation, protein modification and DNA damage (Abdel-Rasol and El-Sayed, 2026). Animal experiments confirm that single or repeated doses of atorvastatin, simvastatin and rosuvastatin reduce plasma and brain glutathione and increase malondialdehyde, accompanied by elevated plasma ALT and AST (Al-Shalchi and Mohammad, 2024). One study provided the first systematic characterization of P450-mediated metabolic activation of atorvastatin: two phase I metabolites (M1, M2) were detected in an NADPH-supplemented system, and the use of glutathione and cysteine as trapping agents revealed four conjugates (M3–M6), indicating the generation of electrophilic quinone-imine intermediates. After intragastric administration to mice (16.4 mg/kg), corresponding glutathione conjugation and protein adduction were observed *in vivo*. CYP3A was the predominant enzyme responsible, and pretreatment with the CYP3A inhibitor ketoconazole significantly reduced both protein adduction and hepatocyte susceptibility to atorvastatin cytotoxicity (Guan et al., 2026). Atorvastatin inhibits the bile-acid transporters BSEP, NTCP and MRP2, leading to bile-acid accumulation and cholestatic injury, which aligns with the cholestatic phenotype observed clinically (Abdel-Rasol and El-Sayed, 2026). In high-fat-diet and apolipoprotein E-deficient mice, a dual GPBAR1/FXR agonist given together with atorvastatin lowered plasma cholesterol and low-density lipoprotein, attenuated liver steatosis and aortic plaque formation, and shifted the bile-acid pool towards FXR antagonists and GPBAR1 agonists (Marchianò et al., 2023). This supports a role for bile-acid signalling in the metabolic and vascular components of NAFLD, but it is animal evidence and does not bear directly on hepatotoxicity. ROS and accumulated bile acids activate Kupffer cells and the NLRP3 inflammasome, amplifying inflammatory cascades including TNF-α and IL-1β (Abdel-Rasol and El-Sayed, 2026). It has been hypothesised that the drug or its metabolites act as haptens that alter hepatocyte antigenicity and trigger immune-mediated injury; this remains a hypothesis derived from experimental work and has not been demonstrated in patients with statin-associated autoimmune-like hepatitis (Serrano-Pérez et al., 2026). Intrinsic antioxidant responses are critical in limiting injury. In cell experiments, atorvastatin exposure upregulated Nrf2 and its downstream target aldo-keto reductase AKR7A2; pharmacological or genetic inactivation of Nrf2 aggravated cytotoxicity, whereas AKR7A2 overexpression attenuated it (Li et al., 2024). Although atorvastatin is a pregnane X receptor (PXR) ligand, its hepatic transcriptomic signature differs substantially from that of the prototypical PXR agonist pregnenolone 16α-carbonitrile. Atorvastatin predominantly activated cholesterol biosynthesis genes, did not induce the classical PXR target Cyp3a11, and did not cause hepatic steatosis, whereas the prototypical agonist aggravated steatosis in high-fat-diet mice. This provides a mechanistic explanation for the relative hepatic safety of atorvastatin in metabolic disease (Nabil et al., 2026). In animal studies, coenzyme Q10 attenuated atorvastatin-induced hepatotoxicity by reducing oxidative stress and restoring CYP3A1 activity from approximately 29% to approximately 90% of control (Abdel-Rasol et al., 2026), and liraglutide mitigated atorvastatin-induced liver injury by restoring GLP-1 receptor expression, activating the Nrf2/SOD pathway, suppressing the HMGB1/TLR-4/RAGE–MAPK/NF-κB axis, and promoting autophagy (Elsiad et al., 2025). Neither agent has been evaluated in patients for this indication, and neither should be presented as a preventive option on the basis of these data.

### 3.6 Inter-statin differences and pharmacogenomic considerations

Using a common data model derived from the electronic health records of approximately 19 million patients across 14 hospitals in South Korea, propensity-score-matched cohorts of between 2,688 and 11,799 patients per group were compared. Moderate-intensity pravastatin 40 mg and moderate-intensity atorvastatin 10 mg were comparably effective for preventing major adverse cardiovascular events, but pravastatin was associated with a lower incidence of abnormal AST elevation (2.35% vs. 3.37%, P < 0.05) and a lower risk of new-onset myalgia or rhabdomyolysis (hazard ratio [HR] 0.82, 95% CI 0.69–0.96) (Chun et al., 2025). A target trial emulation involving 25,728 patients with acute myocardial infarction and elevated liver enzymes in Tianjin, China, found a higher 1-year all-cause mortality with atorvastatin than with rosuvastatin (HR 1.29, 95% CI 1.10–1.51); no significant difference was observed among 18,270 patients with normal liver enzymes (HR 1.09) (Chen et al., 2025). In a propensity-matched cohort of 1,556 patients with primary biliary cholangitis, high-intensity hydrophilic statins (rosuvastatin ≥20 mg) and high-intensity lipophilic statins (atorvastatin ≥40 mg) did not differ in all-cause mortality, major adverse cardiovascular events or hepatic outcomes, but hydrophilic statins were associated with lower risks of all-cause hospitalization (HR 0.595) and cerebral infarction (HR 0.400) (Kawas et al., 2026). A hierarchical risk assessment framework based on pooled Chinese clinical trials from 1992 to 2023 (188 liver-related study units, 31,281 participants) identified pitavastatin as having the highest hepatotoxicity signal (5.36%), atorvastatin as notable for myopathy risk (1.78%), and rosuvastatin and simvastatin as relatively lower risk for both outcomes (Li et al., 2025; Tsui et al., 2025; Tsui et al., 2026). In a comparative HepG2 study, all three statins exerted cytotoxic effects and increased reactive oxygen species production, and these effects were more pronounced with the lipophilic statins atorvastatin and simvastatin than with hydrophilic rosuvastatin, consistent with differences in lipophilicity and intracellular accumulation (Dautović et al., 2023).

### 3.7 Safety and potential benefit in chronic liver disease

In contrast to the pharmacovigilance signals, studies conducted in chronic liver disease consistently support the safety and, in some settings, the benefit of atorvastatin. In a biopsy-proven MASLD cohort of 215 patients (53.5% statin users; simvastatin and atorvastatin accounted for 89.5%; median treatment duration 63 months), statin use was not associated with worsening liver enzymes, and in the subgroup with advanced fibrosis it was not associated with worsening of liver enzymes or liver stiffness (Liou et al., 2026). In a real-world MASLD cohort of 729 propensity-matched patients, atorvastatin 20 mg, rosuvastatin 10 mg and a lovastatin–niacin fixed-dose combination all improved liver biochemistry and lipids, but atorvastatin was associated with more liver enzyme elevation and gastrointestinal intolerance, indicating that the choice of agent should account for individual metabolic and economic factors (Tsai et al., 2026). A randomized, double-blind, placebo-controlled trial in Denmark (n = 78) found that atorvastatin 10–20 mg/day for 6 months was safe in patients with cirrhosis and portal hypertension but did not reduce mortality, liver-related complications or the hepatic venous pressure gradient, with only minor anti-inflammatory effects (Kronborg et al., 2023). A randomized, placebo-controlled trial in Egypt (n = 100) reported that atorvastatin 20 mg/day reduced the cumulative recurrence of cirrhosis-related complications from 72% to 36% (HR 0.50, 95% CI 0.33–0.75, P < 0.001) and completely prevented hepatorenal syndrome (0% vs. 20%). Transaminase elevations were transient and clinically insignificant, and mild myalgia was slightly more frequent (14% vs. 2%) (Glal et al., 2026). The divergence between these two trials may reflect differences in disease stage, dose and endpoint definitions. In a cohort of 329,577 patients with MASLD, statin use was associated with a lower risk of primary liver cancer (adjusted HR 0.64, 95% CI 0.57–0.71) in a dose-dependent manner, with comparable protection for atorvastatin and rosuvastatin (Karachaliou et al., 2026). A meta-analysis of 11 studies found that both lipophilic and hydrophilic statins reduced the incidence of liver cancer (OR 0.54 and 0.56, respectively), with an OR of 0.55 for atorvastatin (Zhang et al., 2024). Cell and computational studies propose mechanisms for this association. Atorvastatin inhibited the proliferation, migration and invasion of hepatocellular carcinoma cells *in vitro* by downregulating the MAPK pathway, although it did not counteract tumour progression in high-fat-diet mice (Meng et al., 2025); lipophilic statins extruded YAP from the nucleus and reduced expression of the YAP/TEAD target genes CTGF and CYR61 in Huh7 and Hep3B cells through Rho GTPase-dependent remodelling of the actin cytoskeleton (Hu and Xiao, 2025); and a network-pharmacology and molecular-docking analysis of 1,948 differentially expressed genes in hepatocellular carcinoma and 380 atorvastatin targets nominated CYP2C9 as the hub gene (Benhammou et al., 2023). These are *in vitro* and *in silico* findings: they support biological plausibility but do not establish that statins prevent or treat liver cancer in patients.A population-based study in Taiwan reported that statins reduced the risk of decompensated cirrhosis in patients with type 2 diabetes and NAFLD in a dose-dependent fashion (HR 0.65, 95% CI 0.61–0.70) (Wu et al., 2023). A meta-analysis of 17 randomized controlled trials (1,344 participants) found that atorvastatin had anti-inflammatory and lipid-lowering effects in patients with pulmonary disease without significant effects on ALT or AST (Baseri et al., 2026), and in a rat model of NAFLD, atorvastatin mainly improved hepatocyte ballooning and lobular inflammation with limited effect on liver enzymes (Eslami et al., 2025). Two further studies address the metabolic rather than the hepatotoxic actions of atorvastatin. In high-fat-diet rats and HepG2 cells, low-dose donafenib combined with atorvastatin activated AMPK signalling, downregulated sterol regulatory element-binding protein-1, HMG-CoA reductase and fatty acid synthase, upregulated fatty acid beta-oxidation proteins, and reduced hepatic oxidative stress and lipid accumulation (Bai et al., 2024). In liver biopsies from 261 patients without known liver disease, statin therapy in those with the metabolic syndrome was associated with higher hepatic transcripts of PNPLA3, acetyl-CoA carboxylase alpha and ATP citrate lyase, and atorvastatin increased PNPLA3 and SREBF2 target genes in hepatocyte-like and hepatic stellate cell models (Ahmed et al., 2025). Neither study was designed to detect liver injury.In a randomized trial of 1,596 individuals followed for 5 years, a fixed-dose combination of aspirin, hydrochlorothiazide, atorvastatin and valsartan reduced major adverse cardiovascular events (HR 0.56, 95% CI 0.41–0.78) and cardiovascular mortality (HR 0.41, 95% CI 0.20–0.86) in patients with MAFLD, with a greater benefit than in the general population (P for interaction 0.028) (Ramandi et al., 2023).

### 3.8 Diagnostic assessment and rechallenge

RUCAM, and its updated version RECAM, remain the most widely applied tools and show good reproducibility in idiosyncratic DILI and DIAIH (Fontana et al., 2023; European Association for the Study of the Liver, 2019). For suspected DIAIH, the simplified autoimmune hepatitis score (S-AIH) may be used as an adjunct, although it was developed for idiopathic autoimmune hepatitis and must be interpreted in clinical context. In one seronegative patient, the diagnosis was ultimately established by liver biopsy together with RUCAM and S-AIH scores of 6 and a clear temporal relationship to the drug (Serrano-Pérez et al., 2026; Singh et al., 2025). The French pharmacovigilance database included 26 cases of atorvastatin-associated liver injury in whom statin therapy was subsequently reintroduced. Rechallenge with the same drug was positive in 12 of 16 cases (11 of 13 at the same dose and 1 of 3 at a reduced dose), and the latency of the recurrent episode was markedly shorter (median 27 days vs. 11 days). Among 10 patients rechallenged with an alternative statin, 2 relapsed (with rosuvastatin and simvastatin), whereas no recurrence was observed in 5 patients receiving rosuvastatin, 2 receiving pravastatin and 1 receiving simvastatin. The authors concluded that rosuvastatin or pravastatin carry the lowest risk of recurrence if lipid-lowering therapy must be continued (Bertin et al., 2026). Additional case reports describe worsening liver injury with concurrent rhabdomyolysis after rechallenge (Singh et al., 2025). Taken together, clinical management can be summarized as structured monitoring, accurate phenotyping, timely discontinuation, cautious switching of agent, and dose reduction in special populations (Table 4).

**TABLE 4:** Clinical management recommendations for atorvastatin-associated liver injury, with the basis and strength of each recommendation.

Stage	Recommendation	Basis and strength	Supporting evidence
Baseline assessment	Measure ALT, AST, total bilirubin, ALP and GGT before starting therapy; assess alcohol use, concomitant medication and underlying liver disease.	Guideline statement (consistent across guidance; not tested in randomised trials)	Fontana et al. (2023); European Association for the Study of the Liver (2019)
Monitoring	Assess liver biochemistry before treatment. No statin-specific randomised evidence defines an optimal surveillance interval, and guidance differs between bodies. Reassessment early after initiation or dose escalation, and then periodically according to baseline risk, is a reasonable framework. Requirements are greater in patients with chronic liver disease, interacting co-medication, or previous clinically significant injury.	Expert opinion (absence of statin-specific randomised evidence; divergent guidance)	US Food and Drug Administration (2012); Fontana et al. (2023); European Association for the Study of the Liver (2019)
Interpretation of an isolated abnormality	Isolated GGT elevation with normal transaminases does not establish hepatotoxicity. Seek alternative causes before attributing the finding to the statin, and avoid unnecessary discontinuation.	Clinical evidence (single case report with confirmatory work-up; low certainty)	Yang et al. (2026)
Discontinuation	Discontinue when ALT or AST exceeds 3 times the upper limit of normal with concurrent bilirubin elevation, when it exceeds 5 times the upper limit of normal, or when symptoms of liver injury are present.	Guideline statement (thresholds adopted from DILI guidance and product labelling)	Fontana et al., (2023); European Association for the Study of the Liver (2019); Abdel-Rasol et al (2026)
Causality assessment	Apply RUCAM or RECAM. For a suspected autoimmune-like phenotype, add S-AIH scoring and consider liver biopsy.	Guideline statement for RUCAM/RECAM; clinical evidence (case reports) for S-AIH in this context; low certainty	Fontana et al. (2023); European Association for the Study of the Liver (2019); Serrano-Pérez et al (2026); Singh et al (2025)
Suspected drug-induced autoimmune-like hepatitis	Withdraw the drug and consider corticosteroid therapy; distinguish from idiopathic autoimmune hepatitis.	Clinical evidence (case reports and case series; low certainty, no controlled data)	Patel et al (2024); Tse et al (2023); Loeza-Suárez et al (2025); Khadijat-Ul-Kurba et al (2025)
Rechallenge	Same-drug rechallenge is not recommended after confirmed injury. If lipid-lowering therapy must continue, prefer a hydrophilic statin, with the lowest observed recurrence reported for rosuvastatin and pravastatin, and monitor closely.	Clinical evidence (pharmacovigilance rechallenge series and case reports; moderate certainty for recurrence, low for agent ranking)	Bertin et al (2026); Singh et al (2025)
Non-statin alternatives	Consider ezetimibe or a PCSK9 inhibitor where statin therapy cannot be continued.	Guideline statement (extrapolated from statin-intolerance guidance rather than from hepatotoxicity data)	Penedones et al (2026)
Choice of statin	Prefer a hydrophilic statin (rosuvastatin or pravastatin) in patients with elevated liver enzymes, chronic liver disease, or previous statin-associated liver injury.	Clinical evidence (observational comparative studies and one target trial emulation; consistent direction, moderate certainty)	Chun et al (2025); Chen et al (2025); Kawas et al (2026)
Chronic liver disease	Do not withhold statin therapy because of concern about hepatotoxicity. In MASLD, compensated cirrhosis and cirrhosis with portal hypertension, the balance of evidence favours treatment.	Clinical evidence (randomised trials and biopsy-proven cohorts; moderate certainty)	Liou et al (2026); Glal et al (2026); Kronborg et al (2023)
Dose adjustment in advanced liver disease	Dose reduction in decompensated cirrhosis should be considered on clinical grounds. The magnitude of reduction cannot be specified from clinical data.	Model-derived only (physiologically based pharmacokinetic simulation, not validated against clinical outcomes); very low certainty	Galal et al (2026)
Pharmacogenomic testing	SLCO1B1 and ABCG2 genotyping may be considered in patients at high risk, but the evidence does not support routine use to guide statin selection.	Expert opinion supported by observational associations only; very low certainty	Alqasrawi et al (2025)

## 4 Discussion

### 4.1 Reconciling strong pharmacovigilance signals with reassuring clinical data

The evidence synthesized here presents an apparent paradox. Atorvastatin accounts for the largest share of statin-associated DILI reports, although its disproportionality signal is exceeded by that of fluvastatin (Yuan et al., 2026; Zhou et al., 2025; Wang et al., 2024), whereas randomized trials and biopsy-proven cohorts show that it is safe and possibly beneficial in MASLD and cirrhosis (Liou et al., 2026; Glal et al., 2026; Kronborg et al., 2023).

Atorvastatin is the most prescribed statin worldwide, so report counts largely reflect the size of the exposed population rather than risk per unit of exposure. Moreover, FAERS analyses compare statins with all other drugs rather than with other statins, and lack denominator data; ROR values therefore cannot be interpreted as absolute risks. The observation that the aHR for generic versus brand atorvastatin converged towards unity as the follow-up period shortened is a textbook illustration of unmeasured confounding (Maruyama et al., 2026). Mild transaminase elevation and clinically significant DILI differ fundamentally in prognosis. Between one-third and one-half of DILI cases in some series present with biochemical abnormality alone (Ma et al., 2024), whereas the outcomes that concern clinicians and patients most—jaundice and liver failure—are extremely uncommon. The apparently favourable hepatic safety profile in chronic liver disease may reflect both altered pharmacokinetics (for example, reduced clearance in cirrhosis producing a mismatch between nominal dose and actual exposure) and differences in immune tolerance (Murphy et al., 2026; Galal et al., 2026). Simple extrapolation of safety data from healthy populations to liver disease populations, or in the reverse direction, is therefore likely to introduce bias.

### 4.2 The autoimmune-like phenotype deserves specific attention

The most important phenotypic development of the past 3 years is the clustering of reports on DIAIH. Its clinical relevance is threefold. First, it is easily misdiagnosed as idiopathic autoimmune hepatitis, delaying withdrawal of the causative drug. Second, it should not be managed as ordinary DILI; the majority of patients require corticosteroids and sometimes immunosuppression. Third, the prognosis is generally good, although treatment may need to be prolonged (Serrano-Pérez et al., 2026; Patel et al., 2024; Tse et al., 2023). Notably, in some cases liver enzymes continued to rise after drug withdrawal and only came under control with immunosuppressive therapy, suggesting that once triggered the immune process becomes relatively autonomous (Khadijat-Ul-Kurba et al., 2025).

### 4.3 From blanket contraindication to risk-stratified prescribing

Earlier guidance was cautious about statin use in chronic liver disease, largely because of hepatotoxicity concerns. The evidence of the past 3 years supports a more granular, risk-stratified approach. In MASLD, compensated liver disease and cirrhosis with portal hypertension, the benefits of statin therapy—cardiovascular protection and potentially anti-fibrotic and chemopreventive effects—usually outweigh the risk of liver injury (Glal et al., 2026; Wu et al., 2023; Karachaliou et al., 2026). In decompensated cirrhosis, however, substantial dose reduction guided by hepatic functional class is required (Galal et al., 2026), and in patients presenting with acute cardiovascular events and already elevated liver enzymes, a hydrophilic statin should be preferred (Chen et al., 2025).

### 4.4 Research gaps

Most mechanistic evidence derives from animal and cell models, and population-level dose–response relationships and safety thresholds remain undefined. Associations with SLCO1B1, CYP3A4/5, UGT1A1 and ABCG2 have been replicated, but prospective randomized trials using genotype to guide statin selection or dosing are still absent (Abdel-Rasol and El-Sayed, 2026; Alqasrawi et al., 2025; Maslub et al., 2024). Current recommendations rest on retrospective pharmacovigilance data and case reports, and there is no consensus on the choice of alternative agent, the timing of rechallenge or monitoring frequency (Bertin et al., 2026). Hepatotoxicity signals for atorvastatin combined with antimicrobial or cardiovascular agents are derived largely from database mining and require confirmation in prospective cohorts or mechanistic studies (Ma et al., 2026). Conventional ALT and AST lack sensitivity and specificity, and the value of novel markers such as microRNAs, glutamate dehydrogenase and keratin-18 fragments in the early recognition of statin-associated liver injury has not been established. An umbrella review of 72 systematic reviews of statin safety rated nearly 86% as critically low in overall confidence, and only 29.2% reported results for individual statins (Penedones et al., 2026). Future work should strengthen design, reporting standards and the presentation of statin-specific data.

### 4.5 Implications for practice

Table 4 summarizes the practical recommendations that follow from the available evidence. In brief, liver function should be assessed before treatment. No statin-specific randomised evidence defines an optimal surveillance interval, and guidance differs between bodies: US product labelling was revised in 2012 to remove the requirement for routine periodic liver enzyme monitoring and to recommend testing before initiation and when clinically indicated (US Food and Drug Administration, 2012), whereas other guidance continues to suggest assessment early after initiation or dose escalation (Fontana et al., 2023; European Association for the Study of the Liver, 2019). The interval proposed below is therefore a practical framework rather than an evidence-based standard, and Table 4 indicates the provenance and strength of each recommendation. Reassessment early after initiation or dose escalation, and then periodically according to baseline risk, is reasonable; the results should be interpreted in the context of injury phenotype and formal causality assessment rather than in isolation; statin therapy should be discontinued when ALT or AST exceeds 3 times the upper limit of normal with concurrent bilirubin elevation, exceeds 5 times the upper limit of normal, or is accompanied by symptoms of liver injury (Fontana et al., 2023; European Association for the Study of the Liver, 2019; US Food and Drug Administration, 2012); same-drug rechallenge should be avoided in confirmed atorvastatin-associated liver injury; and in chronic liver disease, statin therapy should be continued or initiated with dose adjustment rather than withheld.

## 5 Limitations

This review has several limitations. First, only PubMed was searched; Embase, Web of Science, the Cochrane Library and Chinese-language databases were not included, so relevant records may have been missed. Second, the search was built around the term “atorvastatin”; although studies of statins as a class that reported atorvastatin-specific data were included, research reported only under the generic term “statin” may have been underrepresented. Third, the included studies were highly heterogeneous in design, population, dose and outcome definition, precluding quantitative pooling; our conclusions are therefore based on narrative synthesis. Fourth, pharmacovigilance signal values are influenced by reporting bias, duplicate reports and the absence of denominator data and cannot be interpreted as risk ratios. Fifth, although the full text was obtained for all included records, several studies did not state a case definition for liver injury, and this could not be resolved from the published report.

## 6 Conclusion

Evidence published between July 2023 and July 2026 characterizes atorvastatin-associated liver injury as a low-incidence, multi-phenotypic, recognizable event with a high risk of recurrence on rechallenge. Atorvastatin accounts for the largest share of statin-associated DILI reports, and its disproportionality signal is exceeded only by fluvastatin; factors associated with reported injury include dose, age, concomitant medication, underlying liver disease and genetic susceptibility. Mechanistically, mitochondrial dysfunction and oxidative stress, CYP3A-mediated metabolic activation with covalent protein modification, bile-acid transporter inhibition, and immune-inflammatory pathway activation are all implicated. Clinically, particular attention should be paid to recognizing phenotypes such as DIAIH, applying formal causality assessment, avoiding same-drug rechallenge after confirmed injury, and preferring hydrophilic statins or non-statin lipid-lowering strategies when therapy must continue. At the same time, in MASLD, cirrhosis and other chronic liver diseases, statin therapy should not be refused or interrupted because of excessive concern about hepatotoxicity; instead, prescribing and monitoring should be stratified according to hepatic functional class and individual risk.

## Glossary

ABCG2: ATP-binding cassette subfamily G member 2
ADR: adverse drug reaction
AIH: autoimmune hepatitis
AKR7A2: aldo-keto reductase family 7 member A2
ALP: alkaline phosphatase
ALT: alanine aminotransferase
AST: aspartate aminotransferase
ATAC-seq: assay for transposase-accessible chromatin with sequencing
ATO: atorvastatin
ATP: adenosine triphosphate
AUC: area under the plasma concentration–time curve
BSEP: bile salt export pump
CDM: common data model
CI: confidence interval
CYP3A: cytochrome P450 family 3 subfamily A
CYP3A1: rat orthologue of human CYP3A4
CYP3A4: cytochrome P450 family 3 subfamily A member 4
CYP3A5: cytochrome P450 family 3 subfamily A member 5
CYP3A4/5: cytochrome P450 family 3 subfamily A members 4 and 5
Cyp3a11: mouse cytochrome P450 3a11 (classical PXR target gene)
DIAIH: drug-induced autoimmune hepatitis
DILI: drug-induced liver injury
DNA: deoxyribonucleic acid
DOI: digital object identifier
FAERS: US Food and Drug Administration Adverse Event Reporting System
FDA: US Food and Drug Administration
GGT: gamma-glutamyl transferase
GLP-1: glucagon-like peptide 1
GLP-1R: glucagon-like peptide 1 receptor
GSH: glutathione
HepG2: human hepatoblastoma-derived hepatocellular carcinoma cell line
HMG-CoA: 3-hydroxy-3-methylglutaryl coenzyme A
HMGB1: high mobility group box 1
HR: hazard ratio
HVPG: hepatic venous pressure gradient
IL-1β: interleukin 1 beta
IQR: interquartile range
LDL-C: low-density lipoprotein cholesterol
M1: phase I metabolite M1 of atorvastatin
M2: phase I metabolite M2 of atorvastatin
M3–M6: glutathione and cysteine conjugates of atorvastatin metabolites
MACE: major adverse cardiovascular events
MAFLD: metabolic dysfunction-associated fatty liver disease
MAPK: mitogen-activated protein kinase
MASH: metabolic dysfunction-associated steatohepatitis
MASLD: metabolic dysfunction-associated steatotic liver disease
MDA: malondialdehyde
MID-NET: Medical Information Database Network (Japan)
MRP2: multidrug resistance-associated protein 2
NAFLD: non-alcoholic fatty liver disease
NADPH: reduced nicotinamide adenine dinucleotide phosphate
NF-κB: nuclear factor kappa B
NLP: natural language processing
NLRP3: NLR family pyrin domain containing 3
Nrf2: nuclear factor erythroid 2-related factor 2
NTCP: sodium-taurocholate cotransporting polypeptide
OATP1B1/1B3: organic anion transporting polypeptide 1B1 and 1B3
OR: odds ratio
P450: cytochrome P450
PBPK: physiologically based pharmacokinetic (modelling)
PCSK9: proprotein convertase subtilisin/kexin type 9
PMID: PubMed identifier
PXR: pregnane X receptor
Q1: first quarter (of a calendar year)
RAGE: receptor for advanced glycation end products
RECAM: Revised Electronic Causality Assessment Method
ROR: reporting odds ratio
ROS: reactive oxygen species
RUCAM: Roussel Uclaf Causality Assessment Method
|S-AIH: simplified autoimmune hepatitis score
SILI: statin-induced liver injury
SLCO1B1: solute carrier organic anion transporter family member 1B1
SOD: superoxide dismutase
T2DM: type 2 diabetes mellitus
TLR4: Toll-like receptor 4
TNF-α: tumour necrosis factor alpha
UAE: United Arab Emirates
UGT1A1: UDP-glucuronosyltransferase 1A1
ULN: upper limit of normal
US: United States

## References

[B1] Abdel-Rasol M. A. El-Sayed W. M. (2026). Mechanistic insights into atorvastatin-induced hepatotoxicity: molecular pathways, clinical relevance, and strategies for safer personalized therapy. Clin. Exp. Med. 26 (1), 178. 10.1007/s10238-026-02091-w PMC12979359 41784742

[B2] Abdel-Rasol M. A. Anwar M. S. Taha H. A. El-Sayed W. M. AbdelRaouf K. (2026). Coenzyme Q10 protects against atorvastatin-induced hepatotoxicity via attenuation of oxidative stress and functional modulation of CYP3A1. BMC Pharmacol. Toxicol. 27 (1), 63. 10.1186/s40360-026-01125-z PMC13067394 41957824

[B3] Ahmed O. Shavva V. S. Tarnawski L. Dai W. Borg F. Olofsson V. V. (2025). Statin-associated regulation of hepatic PNPLA3 in patients without known liver disease. J. Intern Med. 297 (1), 47–59. 10.1111/joim.20032 PMC11636427 39560367

[B4] Al-Shalchi R. F. Mohammad F. K. (2024). Oxidative stress-induced adverse effects of three statins following single or repetitive treatments in mice. Cureus 16 (1), e51433. 10.7759/cureus.51433 PMC10828976 38298275

[B5] Alqasrawi M. N. Al-Mahayri Z. N. AlBawa'neh A. S. Khasawneh L. Q. Dabaghie L. Altoum S. M. (2025). Pharmacogenomic insights into atorvastatin and rosuvastatin adverse effects: a prospective observational study in the UAE's multiethnic population. Hum. Genomics 19 (1), 44. 10.1186/s40246-025-00753-6 PMC12032684 40281622

[B6] AlTurki A. A. AlQahtani K. Alnasser A. A. AlTalha M. M. AlHamdan A. S. (2026). Statin-induced hyper-acute reversible liver failure: a case report. Cureus 18 (2), e103591. 10.7759/cureus.103591 PMC12990827 41846643

[B7] Bai Y. Chen K. Liu J. Wang Y. Wang C. Ju S. (2024). Activation of AMPK pathway by low-dose donafenib and atorvastatin combination improves high-fat diet-induced metabolic dysfunction-associated steatotic liver disease. Mol. Med. Rep. 29 (3), 51. 10.3892/mmr.2024.13175 38299233

[B8] Baseri S. Izadi M. Alimohammadi M. Khoshnazar S. M. Nikdel R. Hushmandi K. (2026). Effects of atorvastatin on inflammatory markers, lipid profile, liver enzymes, and pulmonary function in patients with lung diseases: a systematic review and meta-analysis of randomized controlled trials. Eur. J. Med. Res. 31 (1), 111. 10.1186/s40001-025-03782-y PMC12825185 41555409

[B9] Benhammou J. N. Qiao B. Ko A. Sinnett-Smith J. Pisegna J. R. Rozengurt E. (2023). Lipophilic statins inhibit YAP coactivator transcriptional activity in HCC cells through Rho-mediated modulation of actin cytoskeleton. Am. J. Physiol. Gastrointest. Liver Physiol. 325 (3), G239–G250. 10.1152/ajpgi.00089.2023 PMC10511177 37366601

[B10] Bertin B. Lacotte V. Cracowski J. L. Gaiffe A. Dumortier J. Vial T. (2026). Atorvastatin-associated liver injury: outcome After statin rechallenge. Fundam. Clin. Pharmacol. 40 (2), e70073. 10.1111/fcp.70073 PMC12906983 41692401

[B11] Chen X. Liu H. Li L. A G. Sun P. Tan D. S. Y. (2025). Atorvastatin versus rosuvastatin in acute myocardial infarction with elevated liver enzymes: a target trial emulation study. Clin. Res. Cardiol. 114 (6), 796–808. 10.1007/s00392-025-02645-0 40208301

[B12] Chun S. W. Kim H. J. Seo J. A. Chon S. Kim S. E. Jung J. H. (2025). Comparative effectiveness and safety of moderate-intensity pravastatin Versus atorvastatin in patients with dyslipidemia: a retrospective cohort study using a common data model of multicenter electronic health records in South Korea. J. Atheroscler. Thromb. 32 (10), 1268–1303. 10.5551/jat.65345 PMC12504035 40350318

[B13] Dautović E. Rustemović-Čorbić M. Srabović N. Softić A. Smajlović A. Mš H. (2023). Some pleiotropic effects of statins on hepatocellular carcinoma cells: comparative study on atorvastatin, rosuvastatin and simvastatin. Adv. Med. Sci. 68 (2), 258–264. 10.1016/j.advms.2023.07.002 37478516

[B14] Elsiad E. A. Abd El Aal H. A. Salem H. A. El-Yamany M. F. Rabie M. A. (2025). Liraglutide Attenuates Atorvastatin-induced hepatotoxicity by restoring GLP-1R expression and activating Nrf2 and autophagy pathways in wistar rats. Toxics 13 (7), 594–611. 10.3390/toxics13070594 PMC12299754 40711038

[B15] Eslami Z. Joshaghani H. Eghbal M. A. Norouzi A. Mirghani S. J. (2025). Atorvastatin and flaxseed dietary treatments improve dyslipidemia and liver injuries in a diet-induced rat model of non-alcoholic fatty liver disease. Avicenna J. Phytomed 15 (3), 1102–1112. 10.22038/ajp.2024.25220 PMC12068500 40365191

[B16] European Association for the Study of the Liver (2019). EASL clinical practice guidelines: drug-induced liver injury. J. Hepatol. 70 (6), 1222–1261. 10.1016/j.jhep.2019.02.014 30926241

[B17] Fontana R. J. Liou I. Reuben A. Suzuki A. Fiel M. I. Lee W. (2023). AASLD practice guidance on drug, herbal, and dietary supplement-induced liver injury. Hepatol 77 (3), 1036–1065. 10.1002/hep.32689 PMC9936988 35899384

[B18] Galal N. El-Khodary N. M. Hussien M. H. A. Abdelkawy K. El-Khateeb E. (2026). Physiologically based pharmacokinetic modeling for optimal dosage prediction of statins and warfarin in patients with liver cirrhosis. Eur. J. Clin. Pharmacol. 82 (4), 106. 10.1007/s00228-026-04027-5 41896467

[B19] Glal K. El-Haggar S. M. Abdel-Salam S. M. Mostafa T. M. (2026). Atorvastatin reduces recurrent decompensation events in advanced cirrhosis in a randomized placebo-controlled trial. Sci. Rep. 16 (1), 9669. 10.1038/s41598-026-41326-4 PMC13009256 41865069

[B20] Guan C. Zhang M. Li Y. Li H. Li W. Hu Z. (2026). Protein covalent modification and hepatic cytotoxicity of atorvastatin resulting from its metabolic activation. Arch. Toxicol. 100 (5), 1927–1940. 10.1007/s00204-025-04222-w 41677906

[B21] Hu Y. Xiao Y. (2025). Mechanism of atorvastatin in treating hepatocellular carcinoma: a study based on network pharmacology, molecular docking, and bioinformatics analysis. Naunyn Schmiedeb. Arch. Pharmacol. 398 (5), 5693–5703. 10.1007/s00210-024-03598-3 39607545

[B22] Karachaliou G. S. Perkins A. M. Dorn C. Reeves R. M. Arnold T. Bashir M. R. (2026). Association of statin use with reduced primary liver cancer risk, independent of age and cirrhosis protection in MASLD. Cancers (Basel) 18 (7), 1132. 10.3390/cancers18071132 PMC13072028 41976355

[B23] Kashyap K. Bisht K. Dhar M. Mittal K. (2023). Atorvastatin-induced Myositis and drug-induced liver injury. J. Assoc. Physicians India 71 (10), 96–98. 10.59556/japi.71.0309 38716533

[B24] Kawas H. Alabbas M. Abu-Hammour M. N. Eren B. Hazem W. Sims O. T. (2026). High-intensity hydrophilic versus lipophilic statins in patients with primary biliary cholangitis: a multi-institutional analysis. BMC Gastroenterol. 26 (1), 236. 10.1186/s12876-026-04723-7 PMC13085312 41803751

[B25] Khadijat-Ul-Kurba H. Zuhra H. Ummul A. O. Nurwahyuna R. (2025). Atorvastatin unveiling primary biliary cholangitis and autoimmune hepatitis overlap syndrome in an older adult: a case report. Malays Fam. Physician 20, 34. 10.51866/cr.853 PMC12258511 40662118

[B26] Kronborg T. M. Schierwagen R. Trošt K. Gao Q. Moritz T. Bendtsen F. (2023). Atorvastatin for patients with cirrhosis. A randomized, placebo-controlled trial. Hepatol. Commun. 7 (12). 10.1097/hc9.0000000000000332 PMC10697620 38051553

[B27] Kuraning K. Ganesh K. M. Arayamparambil P. V. Chandan G. Sarada P. P. (2025). A case report of statin-induced myopathy, rhabdomyolysis and liver injury (SILI): a rare but potentially life-threatening adverse effect. Euroasian J. Hepatogastroenterol 15 (2), 204–207. 10.5005/jp-journals-10018-1488 PMC12932349 41757155

[B28] Li D. Chen J. Zhou F. Zhang W. Chen H. (2024). Aldo-keto reductase-7A2 protects against atorvastatin-induced hepatotoxicity via Nrf2 activation. Chem. Biol. Interact. 393, 110956. 10.1016/j.cbi.2024.110956 38484826

[B29] Li W. Wang D. Lin C. Cai T. Zhao M. Liang L. (2025). A meta-analysis of the incidence of adverse reactions of statins in various diseases. Cardiovasc Ther. 2025, 6684099. 10.1155/cdr/6684099 PMC12173554 40529509

[B30] Liou W. L. Pei Y. Teh K. K. J. Cheah M. C. C. Leow W. Q. Lim T. K. H. (2026). Safety of statins in an Asian cohort with biopsy proven Metabolic dysfunction-associated steatotic liver disease. Dig. Dis. Sci. 71 (8), 3709–3716. 10.1007/s10620-026-09870-1 41915352

[B31] Loeza-Suárez J. García-Luna D. C. Hernández-Mirón J. Ramírez-Marcial R. Valero-Gaona G. A. (2025). Autoimmune Hepatitis (AIH) related to drug-induced liver injury (DILI) due to atorvastatin. Cureus 17 (4), e82315. 10.7759/cureus.82315 PMC12080954 40376367

[B32] Ma X. Chen Z. An J. Zhang C. (2024). Clinical features and risk factors for drug-induced liver injury: a retrospective study from China. Clin. Ther. 46 (8), 597–603. 10.1016/j.clinthera.2024.04.014 38821767

[B33] Ma J. Chen H. Guo C. He G. Yang G. (2026). High-throughput signal detection of hepatotoxic drug-drug interactions in hospitalized elderly patients: an NLP-driven pharmacovigilance study. Ann. Med. 58 (1), 2682581. 10.1080/07853890.2026.2682581 PMC13288716 42311091

[B34] Marchianò S. Biagioli M. Bordoni M. Morretta E. Di Giorgio C. Vellecco V. (2023). Defective bile acid signaling promotes vascular dysfunction, supporting a role for G-Protein bile acid receptor 1/Farnesoid X receptor agonism and statins in the treatment of nonalcoholic fatty liver disease. J. Am. Heart Assoc. 12 (23), e031241. 10.1161/jaha.123.031241 PMC10727350 37996988

[B35] Marušić R. Šojat D. Bačun T. Nešković N. Debeljak Ž. Glegj M. (2026). Impact of SLCO1B1 polymorphism and vitamin D status on statin efficacy and tolerability in postmenopausal women. Biomedicines 14 (1), 113. 10.3390/biomedicines14010113 41595648 PMC12838823

[B36] Maruyama H. Kinoshita Y. Ando T. Okui J. Komamine M. Kajiyama K. (2026). Safety comparison of risk of liver dysfunction between generic and brand Statin drugs marketed in Japan: a cohort study using MID-NET®. Ther. Innov. Regul. Sci. 60 (2), 336–345. 10.1007/s43441-025-00904-w 41455853 PMC12945947

[B37] Maslub M. G. Daud N. A. A. Radwan M. A. Sha'aban A. Ibrahim A. G. (2024). CYP3A4*1B and CYP3A5*3 SNPs significantly impact the response of Egyptian candidates to high-intensity statin therapy to atorvastatin. Eur. J. Med. Res. 29 (1), 539. 10.1186/s40001-024-02109-7 39523378 PMC11552228

[B38] Meng Y. Mo L. Liang T. Mo S. Han C. He Y. (2025). Atorvastatin negatively regulates MAPK pathway *in vitro* to inhibit proliferation, migration, and invasion of hepatocellular carcinoma cells. Sci. Rep. 15 (1), 41279. 10.1038/s41598-025-25040-1 41271824 PMC12639006

[B39] Misra S. Kairi J. K. Kaur M. Sheoron B. Gupta S. Tuteja M. (2026). Trends in drug-induced liver injury (DILI): a case report-based analysis of clinical presentation, severity, preventability, seriousness, and outcomes over the last decade. J. Basic Clin. Physiol. Pharmacol. 37 (4), 315–323. 10.1515/jbcpp-2026-0012 42043569

[B40] Murphy W. A. Sjöstedt N. Mendes M. D. S. Hartauer M. Brouwer K. L. R. Neuhoff S. (2026). Impact of obesity and MASH on zonal hepatocellular statin exposure: pharmacodynamic insights from a permeability-limited multicompartment liver model. CPT Pharmacometrics Syst. Pharmacol. 15 (1), e70138. 10.1002/psp4.70138 41230730 PMC12823464

[B41] Nabil H. Elkhwanky M. S. Hassanen M. Niskanen E. A. Mysore R. Palvimo J. J. (2026). Atorvastatin regulates hepatic transcriptome PXR dependently but distinct from pregnenolone 16α-carbonitrile and does not induce PXR-mediated liver steatosis. Arch. Toxicol. 100 (4), 1443–1463. 10.1007/s00204-025-04280-0 41535588

[B42] Ogura T. Shiraishi C. (2025). Comparative analysis of adverse event profiles among seven statins for hypercholesterolemia management using the United States FDA adverse event reporting system. Cureus 17 (3), e81260. 10.7759/cureus.81260 40291284 PMC12032439

[B43] Patel M. Fattah A. Hussaini H. Maneesha F. Ahmed Z. (2024). Drug-induced autoimmune hepatitis: an unusual adverse event of atorvastatin therapy. Cureus 16 (3), e55809. 10.7759/cureus.55809 PMC10999253 38586640

[B44] Penedones A. Mendes D. Rodrigues L. A. Oliveira T. Alves C. Filipe A. (2026). Safety profile of statins: an umbrella review of systematic reviews and meta-analyses. Eur. Cardiol. 21, e42. 10.15420/ecr.2026.03 42582540 PMC13458407

[B45] Ramandi A. George J. Merat S. Jafari E. Sharafkhah M. Radmard A. R. (2023). Polypill protects MAFLD patients from cardiovascular events and mortality: a prospective trial. Hepatol. Int. 17 (4), 882–888. 10.1007/s12072-023-10542-9 37227560

[B46] Ruiz S. A. Ojeda S. M. Sousa M. J. M. Ferrer R. M. T. Ruiz P. R. Rodríguez SEDP (2026). Latency periods and causality assessment: rethinking atorvastatin-related hepatotoxicity. Rev. Esp. Enferm. Dig. 118 (6), 370–371. 10.17235/reed.2025.11312/2025 40503990

[B47] Serrano-Pérez N. H. Rodríguez-Martínez J. S. Feria-Agudelo S. M. Morales-Leyte A. L. Cordova-Gallardo J. (2026). Drug-induced autoimmune hepatitis due to atorvastatin: a complex clinical case and literature review. Gastroenterol. Rep. (Oxf) 14, goag022. 10.1093/gastro/goag022 41798763 PMC12967216

[B48] Singh H. Khalaf A. Kunkle B. F. Gholami S. Lewis J. H. Rangnekar A. S. (2025). Atorvastatin-induced liver injury with concurrent rhabdomyolysis after a positive rechallenge. ACG Case Rep. J. 12 (1), e01570. 10.14309/crj.0000000000001570 39742213 PMC11688008

[B49] Su F. Y. Li W. H. Zhao X. D. Han Q. Xu X. Geng T. (2024). Risk factors analysis of severe liver injury induced by statins. Horm. Metab. Res. 56 (6), 419–423. 10.1055/a-2210-3395 37956980

[B50] Tsai Y. T. Mudiyanselage S. P. K. Tsai Y. J. Maithreepala S. D. Liu W. C. Ko N. Y. (2026). Real-world comparative outcomes of atorvastatin, rosuvastatin, and lovastatin-niacin fixed-dose combination in metabolic dysfunction-associated steatotic liver disease: hepatic, metabolic, and economic perspectives. Diabetol. Metab. Syndr. 18 (1), 194. 10.1186/s13098-026-02208-x 42265807 PMC13560391

[B51] Tse J. Natla S. Mekala R. Crumm I. Olken M. H. (2023). Atorvastatin-induced autoimmune hepatitis: a case report. Cureus 15 (10), e47807. 10.7759/cureus.47807 38021877 PMC10679798

[B52] Tsui L. Wang D. Fan C. Huang Y. Zhang Z. Fang Z. (2025). Evaluation of statin-induced muscle and liver adverse drug reactions in the Chinese population: a retrospective analysis of clinical trial data from 1992 to 2023. Eur. J. Hosp. Pharm. 33 (1), 50–55. 10.1136/ejhpharm-2024-004352 39922684

[B53] Tsui L. Wang D. Fan C. Shi C. (2026). A hierarchical risk assessment framework for head-to-head comparison of statin safety profiles in Chinese patients. Sci. Rep. 16 (1), 16783. 10.1038/s41598-026-47279-y 41957400 PMC13223228

[B54] US Food and Drug Administration (2012). FDA Drug Safety Communication: Important Safety Label Changes to Cholesterol-Lowering Statin Drugs.

[B55] Wang B. Huang S. Li S. Deng Y. Li Z. Wang Y. (2024). Hepatotoxicity of statins: a real-world study based on the US Food and Drug Administration Adverse Event Reporting System database. Front. Pharmacol. 15, 1502791. 10.3389/fphar.2024.1502791 PMC11747658 39840096

[B56] Wu S. Y. Chen W. M. Chiang M. F. Lo H. C. Wu M. S. Lee M. C. (2023). Protective effects of statins on the incidence of NAFLD-related decompensated cirrhosis in T2DM. Liver Int. 43 (10), 2232–2244. 10.1111/liv.15656 37381761

[B57] Yang J. He Y. Zhang Z. (2026). Isolated gamma-glutamyltransferase elevation during statin therapy: a diagnostic pitfall and a stepwise management framework to prevent unnecessary discontinuation. Clin. Case Rep. 14 (6), e72871. 10.1002/ccr3.72871 42256988 PMC13241699

[B58] Yuan K. Huang Z. T. Liu R. L. Liu Z. W. Lv J. Li C. C. (2026). Major adverse events related to atorvastatin calcium hydrocarbons: pharmacovigilance study in the FAERS database. Can. J. Physiol. Pharmacol. 104, 1–15. 10.1139/cjpp-2025-0288 42091231

[B59] Zhang X. Lou D. Fu R. Wu F. Zheng D. Ma X. (2024). Association between statins types with incidence of liver cancer: an updated meta-analysis. Curr. Med. Chem. 31 (6), 762–775. 10.2174/0929867330666230701000400 37393552 PMC10661961

[B60] Zhou L. Wu B. Bian Y. Lu Y. Zou Y. Lin S. (2025). Hepatotoxicity associated with statins: a retrospective pharmacovigilance study based on the FAERS database. PLoS One 20 (7), e0327500. 10.1371/journal.pone.0327500 40632746 PMC12240319

